# The use of free fibula-flexor hallucis longus osteomuscular flap for calcaneal reconstruction after partial calcanectomy for the chronic osteomyelitis: A case report^☆^^☆☆^

**DOI:** 10.1016/j.ijscr.2019.10.046

**Published:** 2019-10-28

**Authors:** Luís Mata-Ribeiro, Diogo Casal, João Amaral Ferreira, Daniel Sá Costa, João Lacerda

**Affiliations:** a Department of Plastic and Reconstructive Surgery, Hospital São José (Centro Hospitalar Lisboa Central), Lisbon, Portugal; b Department of Plastic and Reconstructive Surgery, Hospital CUF Infante Santo, Lisbon, Portugal; c Anatomy Department, Nova Medical School, Universidade Nova de Lisboa, Portugal; d Department of Orthopaedics, Hospital CUF Infante Santo, Lisbon, Portugal

**Keywords:** CGS, collagen-gentamicin sponges, CO, chronic osteomyelitis, CT, computerized tomography, MSSA, methicillin sensible staphylococcus aureus, MRI, magnetic resonance imaging, Calcaneum, Case report, Fibula, Free flap, Osteomyelitis

## Abstract

**Introduction:**

Calcaneal osteomyelitis poses a tough challenge for any reconstructive surgeon. The eradication of the infection and the reconstruction of the defect are the main goals of treatment.

**Presentation of case:**

We present the case of a 53-year-old male with chronic calcaneal osteomyelitis. The patient was submitted to several bone and soft tissue debridements and twice the application of gentamicin/sulfate implants at the wound bed with no success. He was then submitted to a partial calcanectomy followed by obliteration of the bone defect (4.5 × 2 × 1.5 cm) with a free vascularized fibula-flexor hallucis longus osteomuscular flap (bone dimension:4.5 × 1.5 × 1.2 cm; muscle size: 4 × 1.5 × 0.5 cm). The flap remained viable, and the post-operative period was uneventful. Bone incorporation was evident nine months after the surgery with no signs of recurrent infection or avascular necrosis. Final follow up, at one year, showed an excellent functional result. The patient was able to ambulate without assistance and referred occasional minimal foot pain. Donor site morbidity was minimal.

**Conclusion:**

The authors believe that this osteomuscular flap may be a valuable option to reconstruct calcaneal defects after chronic osteomyelitis.

## Highlights

• Calcaneal osteomyelitis is difficult to eradicate.
• Calcaneal debridement and infection control are critical.
• Osteomuscular flaps offer a stable and effective reconstruction.

## 1 Introduction

Intra-articular fractures of the calcaneus present a challenging management problem, as the lack of soft tissue and the poor vascularization on this weight-bearing surface pose a very high risk of post-operative complications [1,2]. One of the most dreaded complications is chronic osteomyelitis (CO). The calcaneus is involved in 7%–8% of all cases of osteomyelitis [3]. In the adult population, these cases are either post-traumatic, post-surgical, or secondary to chronic ulceration, linked to a neurological disorder or decubitus complications. Hematogenous origin is rare. Optimal therapy for CO should combine the eradication of infection, preservation of bone stock and soft tissues as well as retaining ambulation [4,5]. From the functional point of view, the therapy for CO should rather be bone sparing than radical. Coverage with local and free flaps after complete surgical excision of infected soft tissues and osseous structures is a common approach for the treatment of chronic osteomyelitis [1,6]. We present a patient who had a comminuted calcaneal fracture complicated by osteomyelitis, which we successfully treated with partial calcanectomy and reconstruction with an osteomuscular fibula-flexor hallucis longus free flap.

This work has been reported in line with the SCARE criteria [17].

## 2 Case report

**Fig. 1:**
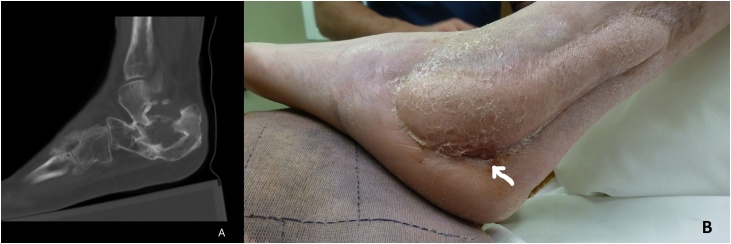
A – Calcaneal osteomyelitis in MRI. B – Pre-operative photograph with sinus tract (arrow).

A 53-year-old male was referred to us four years after suffering a massive trauma of his right foot, sustaining an intraarticular calcaneal fracture treated with internal fixation at the time. Afterward, the patient suffered multiple episodes of local infection, with purulent drainage. The patient was submitted to several calcaneal and soft tissue debridements (twice using a gentamicin sulfate biodegradable implant to fill the bone defect) as well as hardware removal, but the infection recurred every time (osteomyelitis was documented in bone samples). On physical examination, the patient had a small soft tissue deficiency (3 × 3 cm) in the lateral border of the foot and a sinus tract. The MRI confirmed calcaneal osteomyelitis (Fig. 1a). The patient was then submitted to extensive debridement of necrotic bone and the area was covered with a neurocutaneous reverse flow sural flap. Even though the flap was totally viable and healed well, the sinus drainage recurred a few weeks afterward (Fig. 1b).

Several treatment options were discussed (including transtibial amputation) and it was decided to perform a partial calcanectomy and reconstruction with a free osteomuscular fibular flap. Pre-operative angio-CT of the lower limbs showed that the main vascular pedicles were patent on both legs.

**Fig. 2:**
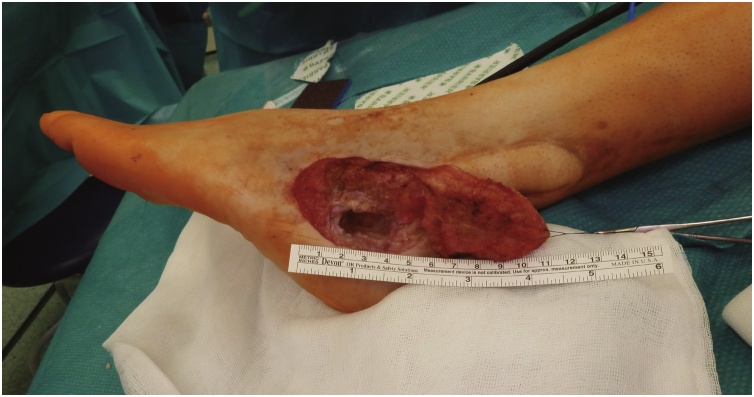
Calcaneal defect after extensive debridement – approximately 4.5 cm of bone gap.

**Fig. 3:**
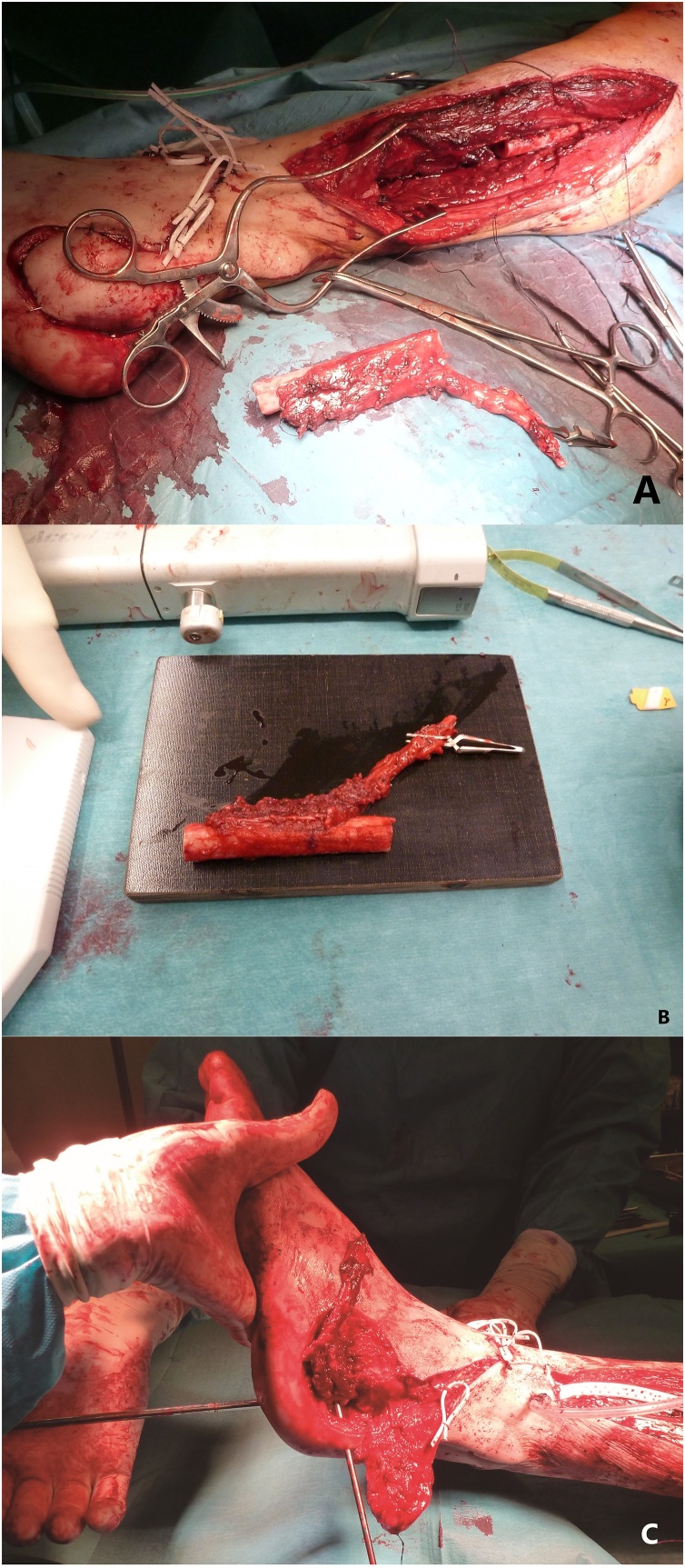
A – Intra-operative photograph after harvest of composite flap. B – Final aspect of the flap just before inset. C – Tibio-tarsal joint fusion.

We conducted an extensive bone debridement to healthy tissue, removing the scar and inflammatory tissue in the vicinity of the wound. This required the removal of a significant amount of calcaneal bone (Fig. 2). Tissue samples (bone and skin) were collected for microbiological analysis. The wound was irrigated with saline and oxygen peroxide. The shape and size for the missing bone were calculated by filling the defect with bone wax and it was approximately 4.5 × 2 × 1.5 cm. After the thorough debridement, the free fibula-flexor hallucis longus was harvested from the same leg (the patient did not want to be operated on the contralateral leg). The dissection proceeded as classically described, but a portion of the muscle belly of flexor hallucis longus was incorporated in the flap. The fibula was transected distally at approximately 6 cm from the lateral malleolus with a vascular pedicle of 7 cm (Fig. 3a). After harvesting the flap, the bone was shaped appropriately with an oscillating saw and a bone rongeur paying particular attention to not harming the main pedicle. The final dimensions of the flap (Fig. 3b) were 4.5 × 1.5 × 1.2 (bone) and 4 × 1.5 × 0.5 cm (muscle component). The flap was inset and stabilized with Kirschner wires to the remaining calcaneus and cuboid bone under fluoroscopy. The Achilles tendon was contracted secondary to several episodes of distal tendinitis and was elongated using a modification of the Hoke method (semi-open triple hemi-section), and the tibiotarsal joint was fused at 90 degrees with the use of a Steinmann pin (Fig. 3c). The peroneal artery was anastomosed end-to-side with the dorsalis pedis artery with interposition of a vein graft, and one of the accompanying veins was anastomosed end-to-end with the lateral marginal vein of the foot. The vein graft was harvested from the great saphenous vein at the distal third of the ipsilateral thigh. Since there was a small skin defect around the area of the interpositional graft, we opted to cover it with a partial thickness skin graft, preventing excessive tension. The donor site was the lower abdomen and the wound closed directly. At the end of the surgery, a foot cast was applied.

**Fig. 4:**
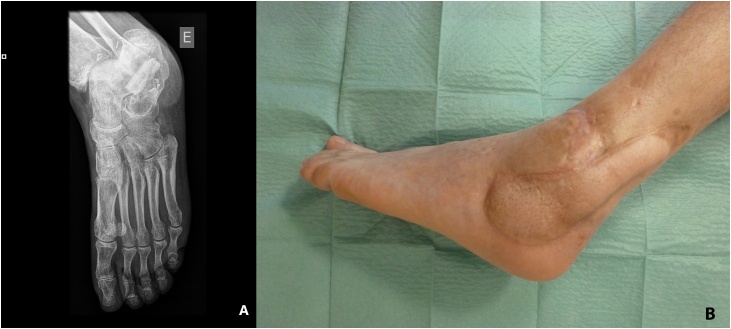
A – Radiographic appearance of the reconstructed defect 9 months postoperatively. B – Photography of the foot 12 months post-operatively.

The post-operative course was uneventful. The bone specimen revealed the presence of Staphylococcus Aureus methicillin sensible (MSSA) and culture-specific antibiotics (ciprofloxacin and clindamycin) were administered intravenously during the hospital stay and orally for eight more weeks. The patient was discharged 16 days after the operation. At ten weeks post-op, the patient was readmitted for the removal of osteosynthesis material. Two weeks later, the cast was removed, and the patient began physical therapy. Nine months post-operatively bone union was evident radiographically (Fig. 4a) with no signs of recurrent infection or avascular necrosis. One year after surgery, the patient could walk without assistance and sustain full weight-bearing (Fig. 4b). The patient started using a personalized calcaneal pad for better contact between the foot and the ground.

## 3 Discussion

Some authors report the successful treatment of calcaneal osteomyelitis with limited debridement and application of special collagen-gentamicin sponges (CGS) in previously drilled holes in the cancellous bone of the calcaneus and with partial calcanectomy and muscle flap coverage or antibiotic cement spacer [2,7,8]. Fraccalvieri et al. demonstrate good outcomes using negative pressure wound therapy, dermal regeneration templates, and skin grafts after partial calcanectomy [9]. Despite this, historically, below-knee amputation or total calcanectomy have been the treatment of choice for extensive osteomyelitic involvement of the calcaneus [1,8,10]. Preservation of a stable, sensate, and plantigrade foot provides better function than does any foot shortening or state-of-the-art prosthesis [11,12]. The use of free muscle flaps is a modern but well-established approach for the treatment of chronic calcaneal osteomyelitis since they can obliterate dead space, improve wound vascularity, increase resistance to bacterial infection, and promote bone healing [11]. However, there are many situations where the extensive debridement leaves a significant bone defect that needs reconstruction.

The application of vascularized bone grafts for correcting both large and small bone defects is widespread [13]. The fibula has specific useful characteristics for this kind of reconstruction namely: sufficient length, can be osteotomized several times without compromising graft viability, and has a high cortical density that enables the reconstructed bone to withstand weight-bearing pressure [11,12]. Nevertheless, most flaps used for reconstruction are just osseous or osteocutaneous and lack the advantages of having a muscular component incorporated in the flap. We overcame that barrier by including a portion of the flexor hallucis longus muscle belly. Lykoudis et al. have used this osteomuscular flap for calcaneal osteomyelitis with a successful outcome [11] as well, and as far as we know, it is the only published paper regarding the treatment of calcaneal osteomyelitis with this flap. Mao et al. reported its use in maxillary reconstruction [14] and Schoeller et al. used it for the functional reconstruction of a complex arm defect after tumor resection [15]. We monitored the flap viability and progress through clinical observation and radiographic evaluation, as described by other authors [11,16]. MSSA was documented in the bone biopsies. This result is consistent with most data published, indicating *Staphylococcus aureus* as the most frequently isolated organism [2].

## 4 Conclusions

Calcaneal osteomyelitis is a severe disease with a high risk of amputation. A complete surgical debridement of all nonviable, infected tissues is necessary for disease control and frequently leaves a substantial defect. We presented a one stage reconstructive approach with a free osteomuscular fibula-flexor hallucis longus flap. This technique allowed a stable bone repair with weight-bearing capacity and ceased the recurrent infectious episodes with minimal donor site morbidity. This technique seems to be a beneficial method for the reconstruction of calcaneal defects, especially when long-standing osteomyelitis is present.

## Source of funding

The authors received no financial support for the research, authorship, and publication of this article.

## Ethical approval

This paper was exempt form ethical approval by the ethics committee of Hospital CUF Infante Santo.

## Statement of informed consent

Written informed consent was obtained from the patient for publication of this case report and accompanying images. A copy of the written consent is available for review by the Editor-in-Chief of this journal on request.

## Author contribution

Luís Mata Ribeiro contributed with the acquisition and interpretation of data, writing and revision of the paper.

Diogo Casal contributed with the concept, writing and final revision of the article.

João Ferreira contributed with the concept, writing and final revision of the article.

Daniel Sá Costa contributed with the concept, writing and final revision of the article.

João Lacerda contributed with the concept, writing and final revision of the article.

All authors read and approved the final manuscript.

## Registration of research studies

This paper does not include a research study.

## Guarantor

Luís Mata Ribeiro (corresponding author).

## Provenance and peer review

Not commissioned, externally peer-reviewed

## Statement of human and animal rights

This article does not contain any studies with human or animal subjects.

## Declaration of Competing Interest

The authors declare no potential conflicts of interest with respect to the research, authorship, and/or publication of this article.
